# Minimally Invasive Strategy to Repair Mitral Valve after Repeated Coronary Revascularization: A Case Report and Literature Review

**DOI:** 10.3390/jcm12227096

**Published:** 2023-11-15

**Authors:** Laura Asta, Umberto Benedetto, Fabrizio Costantino Tancredi, Gabriele Di Giammarco

**Affiliations:** 1 Department of Cardiac Surgery, Tor Vergata University Hospital, 00133 Rome, Italy; 2 Department of Cardiac Surgery, SS Annunziata Hospital, 66100 Chieti, Italy; umberto.benedetto@asl2abruzzo.it (U.B.); fcostantino.tancredi@asl2abruzzo.it (F.C.T.); gabrieledigiammarco57@gmail.com (G.D.G.)

**Keywords:** mitral valve, redo, beating heart, minithoracotomy

## Abstract

Redo cardiac surgery after Coronary Artery Bypass Grafting (CABG) is burdened by high morbidity and mortality, either intraoperatively and postoperatively, with the repeated sternotomy playing a crucial role as risk factor. The right minithoracotomy approach guarantees a safer control on conduits integrity and the right ventricular wall and a low impact on the respiratory mechanics. Herein, we report a patient who previously underwent two CABG (coronary artery bypass grafting) procedures and who was admitted to the hospital with a picture of heart failure caused by a severe mitral regurgitation. He was successfully submitted to a mitral valve repair on a beating heart via the right minithoracotomy approach.

## 1. Introduction

The right minithoracotomy approach for mitral valve surgery constitutes a valid and well-established surgical strategy; however, in our case report, we encourage the use of this approach even in cases of repeated sternotomies. In fact, further sternotomy would have exposed our patient to a high risk of bleeding associated with the presence of adhesions, damage to the myocardium, aorta and lung injury or to the grafts (saphenous vein or mammary artery) previously implanted [1]. Furthermore, the right minithoracotomy has a lower impact on respiratory mechanics, an important aspect in patients with already compromised lung function. Finally, the beating heart technique allowed us to perform the mitral valve repair without handling the aorta, which was difficult to isolate and control. The benefits deriving from this surgical strategy allowed us to obtain a satisfactory result.

## 2. Case Presentation

A 77-year-old man had already undergone two CABG procedures. He received five grafts in 1992 (left anterior mammary artery (LIMA) to left anterior descending (LAD) artery; sequential saphenous vein graft (SVG) to posterior descending artery (PDA), posterolateral (PL) branch, obtuse marginal (OM) and first diagonal artery (D1)). In 2005, the graft failure needed a repeated CABG procedure (right internal mammary artery (RIMA) to anterior descending artery and SVG to PDA). On admission, he complained of dyspnea class IV according to New York Heart Association (NYHA) and signs of severe right heart failure (leg swelling, ascites). The chest x-ray performed on admission showed pulmonary congestion despite medical therapy with furosemide at maximum dosage (500 mg/day) [2].

A Trans-Thoracic Echo2D (TTE) with color flow mapping (CFM) revealed a severe mitral valve regurgitation due to mixed etiology, ischemic cardiomyopathy and mitral valve degeneration. Systemic arterial hypertension, dyslipidemia and hypothyroidism were present in the medical history. In addition, atrial fibrillation treated with non-vitamin K oral anti-coagulants (NOAC), an implantable cardioverter defibrillator (ICD), chronic renal failure (CRF) with creatinine clearance by 20 mL/min and severe chronic obstructive pulmonary disease (COPD) completed the medical history.

**Figure 1:**
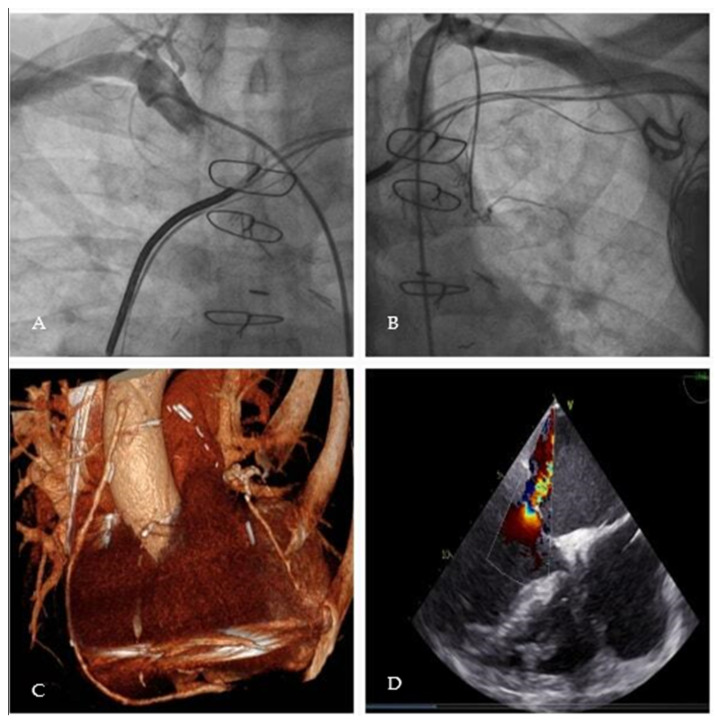
(**A**): RIMA occluded and (**B**): LIMA occluded at coronary angiography; (**C**): SVG to PDA, only patent graft at chest CT scan; (**D**): pre-operative TEE shows a regurgitant jet that reaches the roof of the left atrium (Coanda effect).

As far as cardiac function is concerned, TTE documented a severely depressed left ventricular ejection fraction (LVEF) < 35%, 58 mm of left ventricular end systolic diameter (LVESD), 33 mm/m^2^ of LVESD index (LVESDi) and anterior mitral leaflet (AML) prolapse, causing severe mitral valve regurgitation (MVR). The coronary angiography and chest computed tomography (CT) scan documented total occlusion of all the grafts previously implanted (LIMA, RIMA and sequential SVG) but the most recent (2005) SVG to PDA, which appeared to be a patent with proximal anastomosis positioned very distally to the ascending aorta (Figure 1A–D).

According to the latest guidelines [3] (severe symptoms refractory to medical therapy) and in consideration of the risk of damaging the only patent graft (SVG to PDA), whose course was close to a possible sternal reentry, and pulmonary condition, we chose the right minithoracotomy as the present access route; in addition, we considered the option of continuous coronary perfusion and beating-heart mitral repair to reduce the risk of additional damage from inadequate cardioplegia. Written informed consent for clinical detail and image publication was obtained from the patient.

CPB was established through right femoral vessels (arterial cannula: Optisite 20 Fr, Edwards Lifesciences Italia Spa (Milan, Italy); cavoatrial cannula: HLS 25 Fr, Maquet Cardiopulmonary GmbH (Rastatt, Germany)) and normothermic perfusion. An incision of approximately 6 cm in the right third intercostal space (IS) was performed, and a video-camera (HOPKINS^®^ Telescope 30°, KARL STORZ Endoscopia Italia S.r.l. (Rome, Italy)) port was placed in the fifth IS along the mid-axillary line. The right pleural adhesions were divided, and the pericardium anteriorly exposed the right pulmonary hilum. Once the venous graft to PDA was identified, we proceeded with rapid pacing (150 bpm) and subsequent opening of the left atrium from Soldegar’s sulcus on the beating heart.

Carbon dioxide insufflation at 5 L/min was initiated to prevent air embolism, and venting cannula was placed in the left atrium through the right upper pulmonary vein.

Once the mitral valve was exposed, degenerative sequelae were evident on the AML (A2 scallop) with chordal rupture; a part of the AML and respective chordae was resected. An edge-to-edge repair was performed by suturing A2-to-P2 with Goretex 4/0. Furthermore, an annuloplasty was added using a 32 mm Memo 3D ring (Corcym S.r.l. Saluggia, Vercelli, Italy) secured with 12 Ticron 2/0 U stitches without annular reduction.

**Figure 2:**
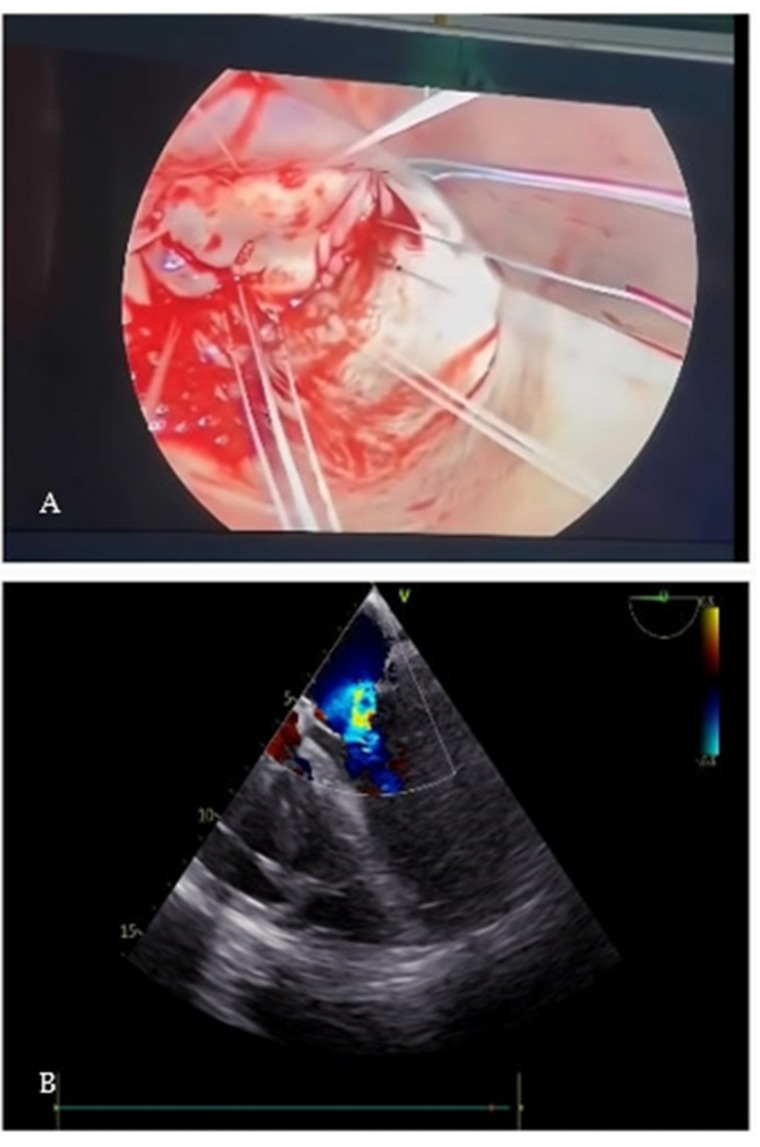
(**A**): Placement of single U-stitches on the native annulus before ring implantation. (**B**): post-operative TEE shows a significantly reduced regurgitant jet.

No injection of saline solution into the left ventricle was used to test the valve after repair to avoid any risk of air or solid particles embolization into the aorta. The left atrium was closed, and the patient progressively weaned from CPB. Transesophageal echocardiography (TEE) showed residual mild-to-moderate mitral regurgitation (Figure 2A,B). The patient was extubated after 24 h. During two days of staying at the intensive care unit (ICU), the rate of inotropic drug was progressively reduced. He was transferred to the ward where he began a respiratory rehabilitation which allowed him to suspend oxygen therapy within a few days. Furthermore, the patient was subjected to gradual weaning from furosemide therapy (from 60 mg/day iv up to 50 mg/day orally). Finally, he was discharged after 10 days without any noteworthy complications.

## 3. Discussion

Although the first surgical approach to the mitral valve involved a thoracotomy access on the left side [4], the median sternotomy was subsequently considered the standard technique to allow for better exposure and cannulation of the great vessels. In 1996, Carpentier presented the first case of video-assisted mitral valvuloplasty performed in a right minithoracotomy [5]. In the following decades until today, minimally invasive techniques up to the robotic technique have experienced rapid growth thanks to satisfactory results obtained. In fact, in addition to a better aesthetic result (not a negligible issue in younger subjects), the right minithoracotomy approach has shown specific advantages: reduction in blood loss, lower rate of infections and pulmonary complications, and reduction in the stroke rate and in-hospital mortality [6,7]. Long- and mid-term outcomes and re-operation rate are substantially comparable to sternotomy access [8].

However, a surgical learning curve has to be considered (75 to 100 cases for a surgeon already an expert in conventional mitral valve surgery); furthermore, the choice of proper candidates, especially in the initial phase, is crucial [9,10].

In addition to the advantages already listed, the right minithoracotomy access has also gained popularity in reoperative cardiac surgery. In fact, re-sternotomy is associated with a high rate of intraoperative complications: bleeding (largely associated with the presence of adhesions), damage to the myocardium (especially atrium and right ventricle), aorta and lung injury or injury to the grafts (saphenous vein or mammary artery) previously implanted [1]. In addition, re-sternotomy is associated with prolonged ICU stay, high rates of blood components transfusion (red cells, fresh frozen plasma, platelets, cryoprecipitate), renal replacement therapy and high mortality [11].

In the case reported herein, the presence of venous and arterial grafts from two previous CABGs operations [12] and the pulmonary conditions (advanced state of COPD) guided the choice of a right minithoracotomy approach. Although arterial grafts were occluded either at coronary angiography and chest CT-scan, it is necessary to stress that in the case where LIMA and RIMA are the patent, the risk of their damage, especially if they cross the midline, is extremely high as is the risk of low cardiac output syndrome due to inadequate myocardial protection [13]. Byrne and colleagues compared the outcomes of 47 patients with patent LIMA-LAD grafts undergoing mitral valve surgery. Thirty-seven patients were approached through a right thoracotomy, with moderate-deep hypothermia and fibrillatory arrest, and 10 were approached through a re-sternotomy, with aortic cross-clamping and cardioplegic arrest. From the comparative analysis, it emerged that patients undergoing right minithoracotomy had a decreased incidence of LIMA-LAD graft injury and, consequently, a lower need for blood transfusions [14]. In addition, this approach can also be used in emergency conditions where the longer re-sternotomy times and the risk of causing damage to previously performed grafts could significantly impact the patient’s outcome [15].

Moreover, the distal position of the SVG proximal anastomosis of the only patent graft (SVG on PDA) on an ascending aorta represented an additional pending risk of possible re-sternotomy.

The temporary single-lung ventilation technique used in the minimally invasive technique is associated with the protection of lung function. This reflects into a reduction in intubation time, ICU stay and, ultimately, a lower rate of pulmonary complications, which is particularly important in our patient who showed a compromised lung function [16,17].

Several studies have shown that right minithoracotomy in a re-intervention is associated with a lower rate of post-operative bleeding, morbidity and in-hospital death [10,18,19].

In particular, Daemen and colleagues demonstrated from their meta-analysis of six retrospective observational studies, enrolling a total of 777 patients, that the rates of mortality, reoperation for bleeding and length of hospital stay were lower in patients undergoing mitral surgery via right minithoracotomy compared to the sternotomy approach [20]. Furthermore, from another meta-analysis conducted by Shirke and colleagues based on the identification of 12 studies, involving 4514 patients, it emerged that the risk of infections and the onset of renal failure requiring dialysis treatment was also lower in patients undergoing mitral surgery for right thoracotomy compared to those subjected via sternal approach. The stroke rate, however, was comparable in the two groups [21].

Therefore, the satisfactory peri- and post-operative clinical results obtained from the treatment of the mitral valve via mini-thoracotomy compared to the traditional median re-sternotomy mean that is today’s minimally invasive approach can be considered an efficient surgical strategy even in re-interventions [22,23].

The difficult isolation and control of the aorta in our patient forced us to avoid cross clamp leading to a beating heart technique; in addition, we feared injury to the vessel after the needle insertion for cardioplegia delivery and aortic vent [24]. Umakanthan and colleagues demonstrated in their observational study on 195 patients undergoing mitral valve repair or replacement surgery that fibrillatory arrest without aortic cross-clamping was not associated with an increase in the risk of stroke nor did it determine a negative impact on the hemodynamic stability of the patients [25]. However, in order for this strategy to be implemented, it is necessary that there is no aortic insufficiency with a degree greater than mild-to-moderate since, in this case, in addition to the difficulty in maintaining a bloodless operating field, it would cause coronary malperfusion.

In the absence of proximal anastomoses on an ascending aorta, the alternative could be the use of the endoaortic balloon. However, its use, certainly safer than in the past, is still linked to a greater risk of aortic dissection and post-operative stroke compared to external aortic cross-clamping [26,27].

We, therefore, believe that “aorta-no-touch” technique was the safest approach [28].

The severe left ventricular dysfunction represented one more reason to choose a beating heart technique with continuous coronary perfusion [29].

Romano and colleagues, in their retrospective study, highlighted that mitral valve repair on a beating heart is associated with shorter cardiopulmonary bypass times, less use of blood and plasma transfusions, shorter intubation time and, consequently, a shorter ICU stay compared to ventricular fibrillation [30]. Furthermore, Pasic and colleagues proved that in heterogeneous patients at high surgical risks and who are not suitable for conventional surgery, mitral valve repair on a beating heart is associated with satisfactory early and midterm results [31].

Therefore, it is possible to state that the combination of the minimally invasive approach and the beating heart technique avoids a prolonged dissection time and leads to significantly shorter operating times and need for transfusion, with all of these issues favoring rapid extubation and a shorter hospital stay [32].

Finally, the edge-to-edge technique allows for a quick repair of mitral regurgitation and, if associated with annuloplasty, guarantees greater durability over time [33]. Some authors do not recommend the beating heart technique when the LAM is involved, as it is believed that repairing maneuvers involving chordae tendineae and anterior papillary muscle is made easier with the heart distended and in cardioplegic arrest [34]. Therefore, a repair technique such as the edge-to-edge one allows for a faster repair without intervening on the chordae tendineae.

In the case reported herein, according to Carpentier’s classification, the mechanism of mitral regurgitation was mixed: excessive leaflet movement (AML prolapse, Carpentier II) and restricted leaflet motion during diastole (tethering of mitral valve leaflets, Carpentier IIIb).

The percutaneous treatment (MitralClip—Abbott) could not be offered to the patient due to the diameters of the left ventricle as LVESD and LVESDi appeared to be exclusion criteria for the use of the device either for immediate or mid-term results. In fact, LVESD > 55 mm (in this case LVESD = 58 mm) is one of the major exclusion criteria for the MitraClip [35]. In addition, some authors have demonstrated that an LVESD ≥ 28 mm/m^2^ (in this case, LVESDi = 33 mm/m^2^) is associated with the worst result in terms of the reduction of mitral regurgitation and a lower reduction in annual hospitalization rate for heart failure compared to patients with LVESDi < 28 mm/m^2^ [36]. Furthermore, although further evaluation trials are still necessary, it would appear that secondary mitral regurgitation (Carpentier IIIb) is associated with a lower cumulative survival and higher rehospitalization and reoperation rate than primary mitral regurgitation (Carpentier II).

Percutaneous mitral annuloplasty using the Carillon™ Mitral Contour System™ (Cardiac Dimension Inc., Kirkland, WA, USA) is a procedure that started in Europe in August 2011, which involves the stabilization of the annulus through coronary sinus (CS) in patients with functional mitral regurgitation [37]. However, primary mitral regurgitation is a contraindication to its use [38], and the trials currently available provide a short follow-up period (<12 months).

Therefore, in our opinion, the surgical choice seemed to be the most appropriate in the case reported herein [39].

## 4. Conclusions

In conclusion, for a beating heart, the right minithoracotomy approach significantly reduced the rate of peri- and post-operative complications in our case and allowed for a successful operation in such a high-risk patient. Furthermore, in consideration of the increasing use of biological prostheses [40] connected to a high rate of structural degeneration and the progressive increase in patients’ age, minimally invasive post-resternotomy techniques need to be further promoted in surgical practice.

## Data Availability

The authors confirm that the data supporting the findings of this study are available within the article.
